# The efficacy of 1% Betadine mouthwash on the incidence of dry socket after mandibular third molar surgery

**DOI:** 10.4317/jced.54444

**Published:** 2018-05-01

**Authors:** Dariush Hasheminia, Amirhossein Moaddabi, Saeid Moradi, Parisa Soltani, Mahsa Moannaei, Maryam Issazadeh

**Affiliations:** 1 Assistant Professor, Department of Oral and Maxillofacial Surgery, School of Dentistry, Isfahan University of Medical Sciences, Isfahan, Iran; 2 Assistant Professor, Department of Oral and Maxillofacial Surgery, School of Dentistry, Mazandaran University of Medical Sciences, Sari, Iran; 3 Dentist, Dental Students Research Center, School of Dentistry, Isfahan University of Medical Sciences, Isfahan, Iran; 4 Postgraduate Student, Department of Oral and Maxillofacial Radiology, School of Dentistry, Isfahan University of Medical Sciences, Isfahan, Iran; 5 Postgraduate Student, Department of Oral and Maxillofacial Radiology, School of Dentistry, Shiraz University of Medical Sciences, Shiraz, Iran

## Abstract

**Background:**

Dry socket or alveolar osteitis is a delayed healing of alveolar bone after exodontia causing moderate to severe pain 2-4 days after extraction of teeth. Antibacterial agents such as antibiotics and chlorhexidine have been previously proved to prevent or reduce the incidence of dry socket. Betadine is a mixture of iodine and povidone which has bactericidal, antifungal and antiviral effects. The aim of the present study was to evaluate the effect of preoperative povidone iodine 1% mouthwash before surgical extraction of impacted mandibular third molar, however age, gender and oral hygiene were also considered.

**Material and Methods:**

189 patients who needed surgical extraction of Pell and Gregory class A and B mandibular third molars were included in this study. The patients who were not willing to participate in the study, took, women who took oral contraceptives or were in the first 22 days of menstrual cycle were excluded. Patients were randomly assigned to control or test group. 97 patients in the test group took preoperative povidone iodine 1% mouthwash and 92 patients in the control group didn’t take any antibiotic or mouthwash. Patients were examined in days 3 and 7 postoperatively for incidence of alveolar osteitis.

**Results:**

Chi-square test didn’t show any significant relation between dry socket incidence and gender (*p* value: 0.848) and Oral hygiene (*p* value: 0.866). However, it revealed a significant relation between age and dry socket incidence (*p* value: 0.003) and patients older than 30 were reported to have higher incidence of dry socket. Independent T-Test showed a significant difference between the test and control group in incidence of dry socket (*p* value: 0.036).

**Conclusions:**

Based on the results of this study povidone iodine 1% mouthwash can decrease dry socket incidence also as the age increases, the incidence of dry socket becomes higher.

** Key words:**Dry socket, impaction, betadine, povidone iodine.

## Introduction

Dry socket or alveolar osteitis was first described at 1896 (1) as a delayed healing process, associated with moderate to severe pain, without any presence of infection, occurring usually two to four days after tooth extraction especially impacted mandibular third molar. Clinical examination reveals an empty socket without any clot or filled with impaired clot and exposed bone surface causing dull and pulsating pain sometimes spreading to the ears, neck and temporal region. halitosis, bad taste in the mouth, presence of necrosis tissue, marginal gingivitis and local lymphadenitis may be present (2). Fever is not common, except in immunocompromised patients or individuals exposed to radiation therapy. Dry socket is self-limiting and not worrisome (3). However, pain can get very severe causing sleep disturbances. Over-the-counter analgesics and narcotics do not appear to reduce the pain (4).

the incidence of dry socket is about 3-5% of all extractions (5,6), but its occurrence after impacted mandibular third molar surgery is between 20% to 35% (7-9).

There has been no definite etiology suggested for dry socket. However, smoking (5), gender (10), the use of oral contraceptives (9), high bacterial load of the mouth before and after the surgery (11,12), tooth position, operator’s skill and experience (5,9), pericoronitis (13) and inadequate irrigation (14) are considered as associated risk factors.

Antibiotics are effective in prevention of dry socket. However, their use is associated with bacterial resistance leading to attempts made in order to find alternative methods with fewer consequences (15).

Betadine or povidone-iodine is a mixture of Povidone and iodine with short acting but wide-spectrum bactericidal effects, sporicidal, fungicidal and virucidal activity as well, used as an antiseptic for infected wounds and also preparing skin and mucous membranes before surgeries. As povidone-iodine solution slowly releases iodine, it is weaker than other products containing free iodine but also less toxic. Betadine leaves no stain, does not cause allergy or tissue irritation and has the longest effect among other antiseptics. Therefore, it is the most commonly used iodinated antiseptic. Recent studies have proved its hemostatic (16) and anti-inflammatory effects (17) as well.

The aim of this study was to evaluate the effect of 1% Betadine mouthwash in prevention of dry socket after surgical extraction of impacted mandibular third molars.

## Material and Methods

189 patients (97 cases, 92 controls) who visited a private dental clinic for surgical extraction of impacted mandibular third molars were included in this study. Inclusion criteria were patients with teeth categorized as class A and B in Pell and Gregory classification (18) not smoking, and not consuming antibiotics or oral contraceptives. Patients signed the informed consent form according to Helsinki Declaration prior to entering in the study. Isfahan Regional Bioethics Committee approved this study (#395189).

The patients were grouped into the experimental and control groups using random odd and even number. The experimental group included patients who were provided with povidone iodine 1% oral antiseptic solution (Betadine, Purdue Pharma L.P., CT, USA), Moreover, gauzes soaked in povidone iodine 1% was placed on the third molars and adjacent teeth for 2 to 5 minutes. Oral rinse was not used for the control group. All patients consumed 400 mg Ibuprofen one hour prior to the operation. During the operation, the intervention for the experimental group involved the use of povidone iodine 1% mouthwash, whereas the control group received no intervention. Afterwards, local anesthesia was carried out with one or two Lidocaine cartridges and epinephrine 1:8000 to induce insensitivity to pain in the inferior alveolar nerve, glossopharyngeal nerve, and long malar nerve. The injection took one minute using the aspiration technique. Surgeries started after a 3-5 minute pause. A sulcular incision from mesial to the second molar continued distally to obtain an inclined incision in the impacted wisdom tooth flap. After elevation of the flap, osteotomy or tooth section was carried out depending on the case and the tooth was extracted. Thereafter, the flap was closed using appropriate sutures. All patients received 400 mg Ibuprofen for three days (every six hours) and instructed to consume medication in case of pain for seven days. Prophylactic or therapeutic antibiotic was not prescribed for any of the patients.

Information was collected using the forms completed prior to operations, examination of patients on the third and seventh postoperative days and filling a questionnaire according to the examination results.

Data was entered to Statistical Package for the Social Sciences (SPSS, IBM, IL, USA) and chi-square test was used for statistical analysis (α=0.05).

## Results

189 patients, 80 men (42.3%) and 109 women (57.7%), requiring surgical extraction of mandibular third molars participated in this study. Dry socket occurred in 18 patients (9.5%), 8 men (10% of total men) and 10 women (9.1% of total women), after the surgery. According to chi-square test, no significant difference was observed between the sexes (*P*>0.05) and also gender distribution of samples in terms of developing dry socket (*P*>0.05) ( Table 1).

**Table 1:**
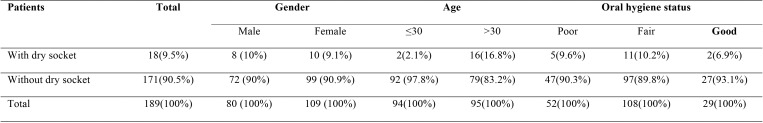
Characteristics of patients with and without dry socket in regard to gender, age, and oral hygiene status.

Age range of the patients was 22 to 46 years old. Mean age of the cases (31.3 years) and the controls (30.4 years) was not statistically different. Chi-square test showed a significant correlation between age and the incidence of dry socket (*P*=0.001). Dry socket was more prevalent among patients older than 30 ( Table 1).

No significant correlation was observed between oral hygiene status and incidence of dry socket (*P*>0.05). There was no significant difference in terms of the oral hygiene at the distribution of patients between test and control groups (*P*>0.05) ( Table 1).

There was a statistically significant correlation between experimental and control groups (*P*=0.036) with the prevalence of dry socket in the experimental group being significantly lower than the controls ( Table 2).

**Table 2:**
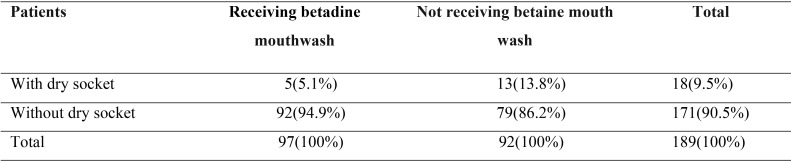
The incidence of dry socket in experimental and control groups.

## Discussion

Findings of the present study indicated that, although incidence of dry socket was not associated to gender, it increased in older patients. Moreover, oral hygiene status of the patients was not correlated to occurrence of dry socket. There was a significant reduction in incidence of dry socket in the experimental group where povidone-iodine 1% oral rinse was used compared to the control group.

Studies report the age related trends in incidence of dry socket. Eshghpour *et al.* (19) in their study reported no significant relationship between incidence of dry socket and age. However, the low number of patients under 26 years can attribute to this lack of significance. Most studies conclude that highest incidence of dry socket is observed in patients between 20 to 50 years of age (20). Incidence of dry socket in the patients under 20 years is very low, which is explained by the better blood circulation, higher elasticity, and the potential for maxillary tissue repair (21). Blondeau *et al.* (22) suggest that surgical exodontia of impacted mandibular third molars should be carried out before the age of 24 years because the likelihood of postoperative complications is higher in older patients. Similar to some other studies, in our research a significant difference between incidences of dry socket was observed in different age groups. Incidence of this condition in people aged over 30 years was considerably higher than people below the age of 30. Since the dry socket condition is an impaired healing condition , the higher incidence of dry socket in older age groups can be explained by the reduced speed and quality of healing.

Cristopher G *et al.* (23) showed that mucosal scar healing is not gender-dependent. This is consistent with the findings of the present study.

Several studies linked poor oral hygiene to incidence of dry socket such as studies performed by Hassan Momeni *et al.* (24), Oginni *et al.* (25) introduced satisfactory oral hygiene as a factor involved in decreased incidence of dry socket. In our study, there was no significant relationship between incidence of dry socket and oral hygiene. Seemingly, the use of the povidone iodine mouthwash exactly before the surgery can influence oral hygiene in more than half the study population. Hence, another study with a larger sample is recommended to further examine the effect of oral hygiene on dry socket.

Rodriguez *et al.* (26) induced bacteria in rats to study the effects of microorganisms and bacterial contamination on escalation of incidence of dry socket. They reported that in order to prevent incidence of dry socket the surgeon must minimize bacterial contamination. Use of different forms of chlorhexidine was found to be effective in reducing the incidence of dry mouth in other papers (2,27).

As compared to chlorhexidine, few studies have been conducted on the effect of povidone iodine on incidence of dry socket (28).

Mesgarzadeh *et al.* (29) studied 277 teeth extracted from 199 patients and introduced the povidone iodine 1% mouthwash as a cause of decreased incidence of dry socket. In our research, there was a significant relationship between the use of the povidone iodine 1% mouthwash and incidence of dry socket and it was found that unlike the findings reported by Sweet *et al.* (30) regarding the lack of effect of antibacterial iodinated solutions on the decrease in incidence of dry socket, this mouthwash can considerably reduce incidence of dry socket. The decrease in incidence of dry socket after application of a topical antimicrobial substance is reflective of the role of bacteria in development of dry socket and indicates that microbial factors could be involved in outbreak of dry socket.

## Conclusions

Under the conditions of the present study povidone iodine 1% mouthwash reduces incidence of dry socket following surgical extraction of impacted mandibular third molars. This finding suggests the use of povidone-iodine prior to surgical extraction of teeth.
